# Modified Simon’s minimax and optimal two-stage designs for single-arm phase II cancer clinical trials

**DOI:** 10.18632/oncotarget.26981

**Published:** 2019-07-02

**Authors:** Jongphil Kim, Michael J. Schell

**Affiliations:** ^1^ Department of Biostatistics and Bioinformatics, Moffitt Cancer Center and Research Institute, Tampa, FL, USA; ^2^ Department of Oncologic Sciences, University of South Florida, Tampa, FL, USA

**Keywords:** admissible two-stage design, binary endpoint, modified two-stage design, Simon’s two-stage design, single-arm phase ii clinical trials

## Abstract

Simon’s two-stage design and the admissible two-stage design have been commonly used in practice for single-arm phase II clinical trials when the primary endpoint is binary. The ethical benefit of the two-stage design over the single-stage design is attained by the early termination of the trial when the treatment seems to be inactive. While Simon’s optimal design is the two-stage design that minimizes the expected number of subjects under the null hypothesis, the probability of falsely declaring futility after the first stage frequently seems undesirably high. In Simon’s minimax design, however, it is often the case that a high proportion of the total planned subjects are evaluated in the first stage, and thus the ethical benefit may not be achieved. In this paper, we propose modified minimax and optimal two-stage designs which guarantee not only type I and II error rates but also reasonable sample size proportions in the first stage, while maintaining the probability of falsely declaring futility under a pre-selected level. The characteristics of the modified two-stage design will be compared with those of Simon’s and the admissible two-stage design. The modified minimax design yields a design that requires modest increase in 29% of cases, while the modified optimal design saves 1 to 13 subjects in 81% of cases for β = 0.2. The modified design approach provides investigators with an alternative when the sample sizes of Simon’s designs are severely unbalanced or the Type II error is unacceptably high after the first stage.

## INTRODUCTION

The primary objective of phase II cancer clinical trials is to seek an early indication of anti-tumor activity of a novel treatment and to make a “go/no-go” decision for a larger and more definitive phase III trial. Although the Clinical Trial Design Task Force (CTD-TF) of the National Cancer Institute (NCI) Investigational Drug Steering Committee (IDSC) in general recommended the use of progression-free survival as the primary endpoint and randomization, the CTD-TF acknowledged that the objective response rate as an endpoint and single-arm designs remain relevant in certain situations (Seymour *et al*. [1]) and such designs remain very common.

The two-stage design for a single-arm phase II clinical trial with binary endpoint has a history dating back to Gehan [2]. The ethical benefit of the two-stage design over the single-stage design is attained by the early termination of the trial when the treatment seems to be inactive. Simon’s two-stage design [3] has been commonly used in practice for single-arm phase II cancer clinical trials when the primary endpoint is binary. Within the framework of two-stage design, the trial will be early terminated if *n_1_* subjects are evaluated in the first stage and the number of responders is less than or equal to *r_1_*. If the trial proceeds to the second stage, then a total of *n* subjects will be evaluated and the null hypothesis fails to be rejected if *r* or fewer responders are observed.

A substantial amount of work has been published concerning two-stage designs with binary endpoint. Herndon [4] proposed a hybrid two-stage design which allows the continuation of patient accrual while the first stage data is being analyzed. Ye and Shyr [5] provided a balanced two-stage design which seeks to equalize the sample size of the two stages, while maintaining total sample size that are comparable with Simon’s design. This design is, however, not an optimal design in terms of either total sample size *n* or the expected sample size under the null hypothesis. Chi and Chen [6] proposed a two-stage design which allows early termination for efficacy and futility. The two-stage adaptive designs by Banerjee and Tsiatis [7] and Lin and Shih [8] and the Bayesian two-stage designs by Heitjan [9], Sambucini [10], Tan and Machin [11], and Wang et al. [12] were developed. The two-stage optimal design for phase II trials under the alternative hypothesis was presented by Mander and Thompson [13].

These design approaches except Ye and Shyr [5], however, do not take into account the balance in sample size between the two stages, and thus the ethical benefit expected by the two-stage design approach may not be achieved if a high proportion of subjects are evaluated in the first stage. In addition, authors have observed the two-stage design of which the probability of falsely declaring futility assigned at the first stage is undesirably high, as the design does not place an upper limit on it. Moreover, no admissible design exists if the difference in *n* between two Simon’s designs is less than or equal to 1. To address these two concerns, we propose modified minimax and optimal two-stage designs which can guarantee not only type I and II error rates but also a reasonable range of sample size of the first stage, while maintaining the probability of falsely declaring futility after the first stage under a pre-selected level.

## METHODS

### Simon’s and the admissible two-stage designs

Suppose that *p_0_* and *p_1_* are the success rates under the null and alternative hypotheses, respectively. For given type I and II error rates of α and β, Simon’s minimax two-stage design is the design, (*r_1_, n_1_, r, n*), which minimizes the total sample size *n*. If multiple solutions, (*r_1_, n_1_, r, n*), exist, the design with the minimal expected sample size under the null hypothesis,

$$EN_{0}=n_{1}\text{+(1}\;\text{-}\;PET_{0}\text{)}\times \text{(}n\;\text{-}\;n_{1}\text{),}$$

is selected as the minimax two-stage design. Herein, the *PET_0_* is the probability of early termination under *p_0_* after the first stage;

$$PET_{0}\;\text{=}\;\text{B(}r_{\text{1}}\;\text{|}p_{0}\text{,}\;n_{1}\text{),}$$

where B(·|*p*, *m*) is the cumulative distribution function for the binomial distribution with success probability of *p* and number of trials, *m*, respectively. Likewise, Simon’s optimal two-stage design is the design which minimizes the *EN_0_* with the same constraints used for the minimax design. The optimal design is a two-stage design for which the *PET_0_* should be as high as possible and *n_1_* as small as possible. Accordingly, the probability of early termination under *p_1_* (*PET_1_*) which corresponds to the type II error spent at the first stage, could be undesirably high, especially for β = 0.2.

The admissible two-stage design by Jung et al. [14] is the design which minimizes the Bayes loss or risk function,

$$q\times n\;+\;\left(\text{1}\;\text{-}\;q\right)EN_{0},\;q\;\in \;\left[0,\;\text{1}\right],$$

with the same constraints as used in Simon’s design. Simon’s minimax and optimal designs are equal to the admissible two-stage designs with *q* = 1 and *q* = 0, respectively. Thus, no additional admissible design exists if the difference in *n* between two Simon’s designs is less than or equal to 1.

As these Simon’s designs and the admissible designs do not take into account the balance in the sample size and type II error between two stages, the severe imbalance in the sample size or in type II error is often observed. For example, with design parameters (*p_0_*, *p_1_*, α, β) = (0.7, 0.9, 0.05, 0.2), 23 of 26 (88%) and 6 of 27 (22%) subjects will be evaluated in the first stage by Simon’s minimax and optimal designs, respectively, and no additional admissible two-stage design is available. The type II errors spent in the first stage by Simon’s minimax and optimal designs are 19.3% and 11.4%. For Simon’s minimax design with design parameter (*p_0_*, *p_1_*, α, β) = (0.5, 0.65, 0.05, 0.2), 66 out of 68 subjects (97%) will be evaluated in the first stage, while Simon’s optimal design requires additional 15 subjects. The type II error spent at the first stage by Simon’s minimax and optimal designs are as high as 18.9% and 14.3%. Other examples will be discussed in Section 3.3.

### Modified minimax and optimal two-stage designs

We propose the modified minimax two-stage design for single-arm phase II clinical trials which is the solution, (*r_1_, n_1_, r, n*), to an integer optimization problem expressed by

minimize *n*

$$\text{subjects}\;\text{to}\;\;PET_{1}\;=\;B(r_{1}|p_{1},\;n_{1})\;\leq \;\varepsilon \;\leq \;\;\text{β},$$ (1)

$$\lambda_{\text{1}}n\leq n_{1}\leq \;\lambda_{\text{2}}n,\;0\;\text{<}\;\lambda_{\text{1}}\;<\;\lambda_{\text{2}}\;\text{<}\;\text{1},$$ (2)

Type I error ≤ α and Type II error ≤ β.

The aforementioned two drawbacks of Simon’s design can be addressed by considering two additional constraints (1) and (2). With appropriate values of λ_1_, λ_2_, and ε, the pre-selected range of subjects will be evaluated in the first stage and the probability of falsely declaring futility spent at the first stage will be less than or equal to ε ≤ β. As ε, a maximally allowed type II error at the first stage, gets close to β, the impact of constraint (1) becomes diminished. Likewise, the modified optimal two-stage design is the solution which minimizes *EN_0_* with the same constraints. Note that the modified two-stage design matches Simon’s design if it satisfies equation (1) and (2). Investigators may choose different values of λ_1_, λ_2_, and ε, depending on their purpose. λ_1_ = 1/4 and λ_2_ = 1/2, for instance, could be selected if one wants to conduct the interim analysis with 25% to 50% of the planned information for whether the second stage is open. The optimal timing for interim analyses for the confirmative clinical trials has been examined by Lawrence Gould [15] and Togo and Iwasaki [16]. Lawrence Gould claimed that the interim analysis for futility for randomized two-arm ‘proof of concept’ trials be carried out after accumulating at least 40% of the planned observations. As Lawrence Gould pointed out, if the interim analysis for futility is carried out with too little data, it is not conclusive enough to support the decision. Little benefit will be gained if the interim analysis is conducted with too much data. In this paper, λ_1_ = 1/3, λ_2_ = 2/3 and ε = 0.1 are selected to provide practical boundary so that 33% to 67% of subjects will be evaluated in the first stage to make decision with the reasonable amount of data and the *PET_1_* is controlled under 0.1. For β ≤ 0.1, constraint (1) makes no impact on searching for the solution. With constraint (1), the modified design, however, guarantees that when β is chosen to be > 0.1, the probability of falsely declaring futility after the first stage is controlled to be at most 10%. For β =0.2, a common choice, the modified design is well balanced in terms of type II error as well as sample sizes between two stages. Simon’s and the admissible design were computed through Dr. Anastasia Ivanova’s website [17].

## RESULTS

### Comparisons with Simon’s and the admissible design

**Figure 1:**
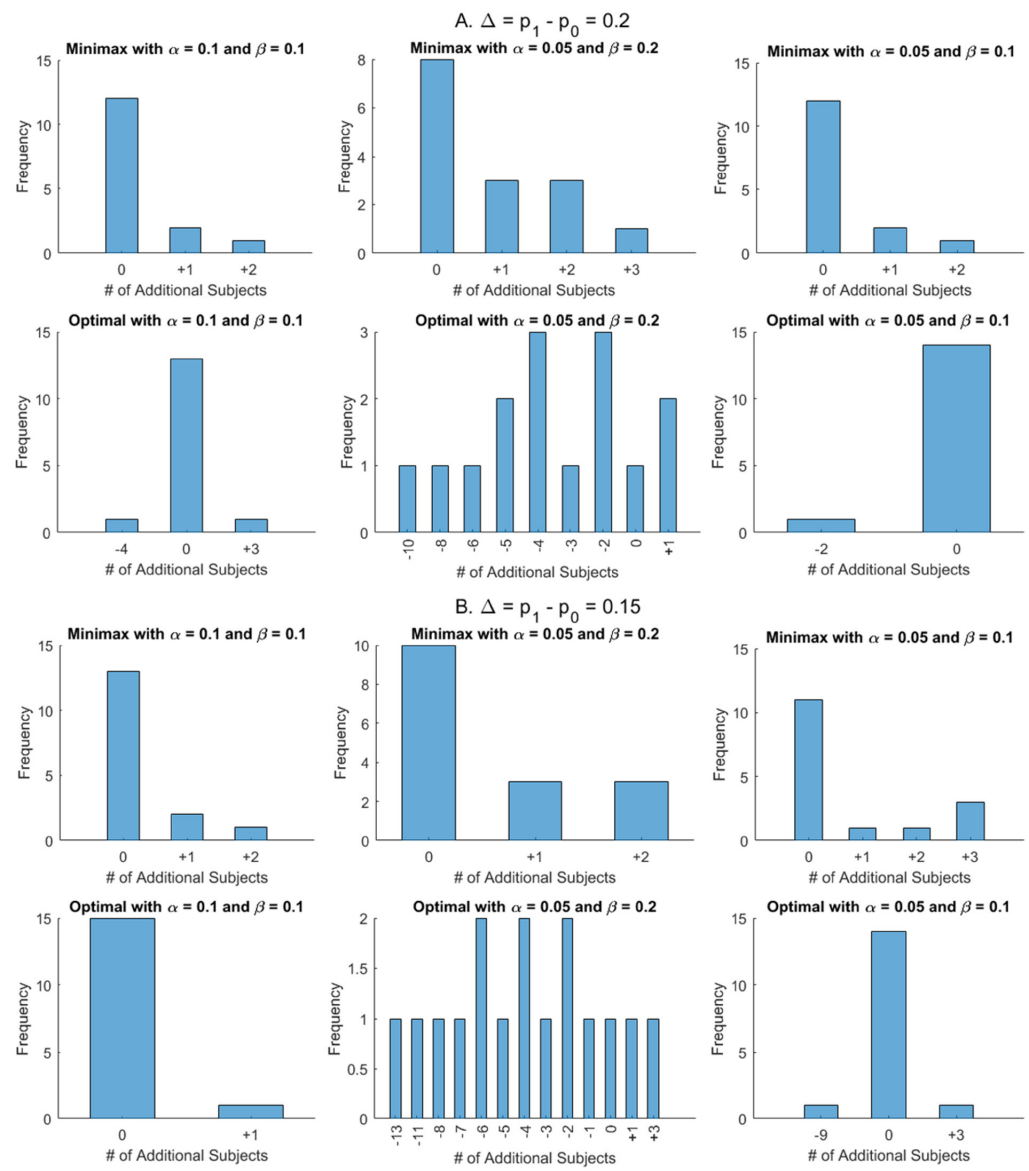
Comparisons of total sample sizes between modified designs with γ_1_ = 1/3, γ_2_ = 2/3, and ε = 0.1 and Simon’s designs for p_1_ – p_0_ = 0.2 (**A**) and p_1_ – p_0_ = 0.15 (**B**). The top panels of A and B show the number of additional subjects required for the modified minimax designs while the bottom panels indicate those for modified optimal designs.

Firstly the total sample size of the modified design with γ_1_ = 1/3, γ_2_ = 2/3, and ε = 0.1 is compared with Simon’s design for Δ = *p_1_* - *p_0_* = 0.15 (16 cases) and 0.2 (15 cases) in Figure 1. The top panels of Figure 1A and 1B show the number of additional subjects required for the modified minimax design while the bottom panels indicate those for the modified optimal design. Overall, 66 of 93 (71%) have the same total sample size to Simon’s design (10 (11%) have different first stage numbers), with the remaining 27 cases (29%) needing at most 3 additional subjects. For the modified optimal design, the results differ dramatically by β. For β = 0.1, 56/62 cases (90%) have the same total sample size, while 3 cases each require more (1 to 3 subjects) or fewer (2 to 9 subjects). For β = 0.2, only 2 cases (6%) have the same total sample size, while 81% (25/31) of cases save 1 to 13 subjects, and 13% (4/31) require 1-3 additional cases. Thus, for β = 0.2, dramatic improvements over the Simon design can be achieved.

The further comparisons are conducted and summarized in Supplementary Figures 1 and 2. The number in parenthesis for (α, β) = (0.05, 0.2) denotes the difference in *n*, compared with the modified optimal design. In cases that there is no difference in *n*, we investigate if the sample size of the first stage *n_1_* and the early stopping rule for futility *r_1_* are identical; “=” indicates that the designs are identical but “≠” shows that they are not identical even though the total sample sizes are the same. For example, for (*p_0_*, *p_1_*, α, β) = (0.3, 0.5, 0.1, 0.1), the modified minimax design is not identical to Simon’s minimax even though the total sample sizes are the same; (*r_1_*, *n_1_*, *r*, *n*) = (6, 26, 15, 39) for the modified minimax against (7, 28, 15, 39) for Simon’s minimax. For (*p_0_*, *p_1_*, α, β) = (0.05, 0.25, 0.05, 0.2), three designs, the modified minimax and optimal and Simon’s optimal design ((*r_1_*, *n_1_*, *r*, *n*) = (0, 9, 2, 17)), are identical, and one more subject is required in *n*, compared to Simon’s minimax design, (*r_1_*, *n_1_*, *r*, *n*) = (0, 12, 2, 16).

**Figure 2:**
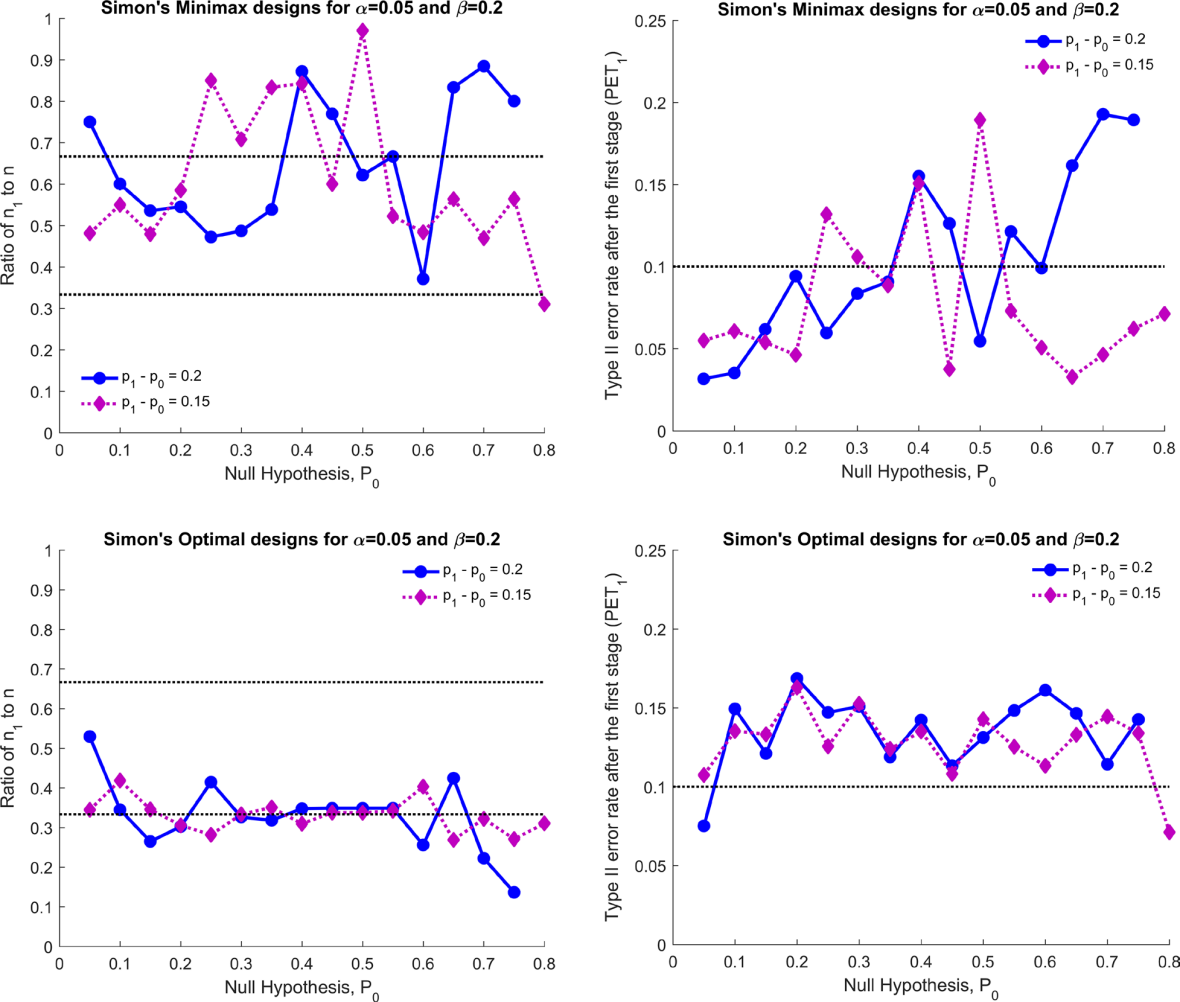
Simon’s minimax (top two panels) and Simon’s optimal designs (bottom two panels) for *p_1_* - *p_0_* = 0.15 and 0.2: ratio of *n_1_* to *n* (left panels) and the type II error spent in the first stage (*PET_1_*, right panels) for (α, β) = (0.05, 0.2).

Figure 2 illustrates the characteristics of Simon’s designs for (α, β) = (0.05, 0.2). The left and right panels show the ratio of *n_1_* to *n* and the Type II error rate spent after the first stage (*PET_1_*), respectively. Top and bottom panels show Simon’s minimax and optimal designs, respectively. The *PET_1_* of Simon’s minimax design is greater than 0.1 in 10 of 31 cases (32%) and less than *n*/3 subjects will be investigated in either the first or the second stage in 11 of 31 cases (35%). The *PET_1_* of Simon’s optimal design for (α, β) = (0.05, 0.2) is greater than 0.1 except for two cases, *p_1_* - *p_0_* = (0.05, 0.25) and (0.8, 0.95). With (α, β) = (0.1, 0.1) and (0.05, 0.1), all *PET_1_*s of Simon’s minimax and optimal design considered satisfy constraint (1) (plots are omitted) and thus the modified designs are not identical to Simon’s designs if <*n*/3 subjects are evaluated in either the first or the second stage; 24 of 62 (39%) for Simon’s minimax and 9 of 62 (15%) for Simon’s optimal design. The *EN_0_* of the modified minimax design is smaller than or equal to Simon’s minimax except for 4 cases (plots are omitted) while the *EN_0_* of the modified optimal design increases by 0.04 to 3.36. As the *EN_0_* is highly attributed to the sample size in first stage, *n_1_*, the large difference in *EN_0_* between the modified and Simon’s design can be found when the ratio of *n_1_* to *n* is too large or too small.

### Examples

The characteristics of the modified design are compared in detail with the other two designs in Table 1 for four cases. With (*p_0_*, *p_1_*, α, β) = (0.35, 0.55, 0.1, 0.1), 86% of subjects will be evaluated in the first stage by Simon’s minimax design while 48% will be evaluated in the first stage by the modified minimax design. The modified minimax design is identical to an admissible design and requires two additional subjects in *n*. The *EN_0_* of the modified minimax, however, decreases by 5.2. The modified optimal design is the same as Simon’s optimal design.

**Table 1:** Comparisons of the characteristics of the modified designs to Simon’s and admissible designs

*p_0_*	*p_1_*	α	β	Design method	*r_1_*	*n_1_*	*r*	*n*	*EN_0_*	*PET_1_*	*n_1_/n*(%)	Comments
0.35	0.55	0.1	0.1	Simon’s Minimax	15	**36**	18	**42**	36.9	0.075	**85.7%**	
				Simon’s Optimal	7	20	20	47	30.8	0.058	42.6%	
				Modified Minimax	7	21	19	44	31.7	0.038	47.7%	Admissible
				Modified Optimal	7	20	20	47	30.8	0.058	42.6%	Simon’s optimal
				Admissible	7	21	19	44	31.7	0.038	47.7%	q ∈ [0.2286, 0.7249]
0.7	0.9	0.05	0.2	Simon’s Minimax	19	**23**	21	**26**	23.2	0.193	**88.5%**	
				Simon’s Optimal	4	**6**	22	**27**	14.8	0.114	**22.2%**	
				Modified Minimax	8	11	23	28	16.3	0.090	39.3%	
				Modified Optimal	8	11	23	28	16.3	0.090	39.3%	
0.8	0.95	0.1	0.1	Simon’s Minimax	5	**7**	27	**31**	20.8	0.044	**22.6%**	
				Simon’s Optimal	5	**7**	27	**31**	20.8	0.044	**22.6%**	
				Modified Minimax	13	16	27	31	21.3	0.043	51.6%	
				Modified Optimal	13	16	27	31	21.3	0.043	51.6%	
0.5	0.65	0.05	0.2	Simon’s Minimax	39	**66**	40	**68**	66.1	0.189	**97.1%**	
				Simon’s Optimal	15	28	48	83	43.7	0.143	33.7%	
				Modified Minimax	20	41	41	69	55.0	0.024	59.4%	Admissible
				Modified Optimal	15	29	44	75	45.4	0.098	38.7%	
				Admissible	20	41	41	69	55.0	0.024	59.4%	*q* ∈ [0.7716,0.9174]
				Admissible	18	35	42	71	48.2	0.068	49.3%	*q* ∈ [0.5151,0.7715]
				Admissible	16	31	43	73	46.1	0.087	42.5%	*q* ∈ [0.285,0.515]
				Admissible	14	27	45	77	44.5	0.111	35.1%	*q* ∈ [0.1189,0.2849]

With (*p_0_*, *p_1_*, α, β) = (0.7, 0.9, 0.05, 0.2), the sample size of each stage for both Simon’s minimax and optimal design is seriously imbalanced (88% and 22% in the first stage) and the *PET_1_*s of them are as high as 19% and 11%. No additional admissible design is available. The modified minimax and optimal design provides investigators with a novel design, (*r_1_, n_1_, r, n*) = (8, 11, 23, 28) which requires 1 or 2 additional subjects if the second stage is open. The *PET_1_* of this design decreases to 9% (approximately 10% and 2% lower than Simon’s optimal and minimax) and 39% of subjects will be evaluated in the first stage.

With (*p_0_*, *p_1_*, α, β) = (0.8, 0.95, 0.1, 0.1), Simon’s minimax design is identical to Simon’s optimal design and 7 of 31 (23%) subjects will be evaluated in the first stage and no additional admissible design is available. Similarly, the modified minimax design is optimal in term of *EN_0_* in those satisfying constraint (1) and (2), and 16 out of 31 (52%) subjects will be evaluated in the first stage. When compared to Simon’s optimal design, the *EN_0_* of the modified design increases by 0.5, which seems ignorable. In fact, the *PET_0_* of the modified design is much higher than that of Simon’s design (0.648 vs. 0.423) and the sample size of the modified design is much better balanced.

With (*p_0_*, *p_1_*, α, β) = (0.5, 0.65, 0.05, 0.2), the sample size of each stage for Simon’s minimax design is severely imbalanced (97% in first stage) and the *PET_1_*s of Simon’s designs are as high as 19% and 14% for Simon’s minimax and Simon’s optimal designs. The sample size of each stage for the modified design is well balanced and the *PET_1_*s are controlled to be below 10%. The total sample size of the modified optimal design decreases by 8, compared with Simon’s optimal but the *EN_0_* of the modified optimal design increases by 1.7. The modified minimax design is identical to one of 4 other admissible designs.

## DISCUSSION

As both Simon’s two-stage designs and the admissible two-stage design approaches do not take into account the balance in the sample sizes between the two stages, a high proportion of subjects may be evaluated in the first stage, and so the ethical benefit expected by the two-stage design is not be achieved. In addition, the Type II error spent at the first stage is frequently undesirably high, as it is not controlled within framework of Simon’s design. We believe that such designs may not be very acceptable to investigators. Moreover, the admissible design does not exist if the difference in total sample size between Simon’s optimal and minimax designs is ≤ 1. These drawbacks of Simon’s design can be improved by using the modified design approach presented here which aims to find the minimax and optimal two-stage design satisfying two additional constraints: 1) reasonable sample size proportion in the first stage and 2) ensuring a Type II error of ≤ ε ≤ β after the first stage. With λ_1_ = 1/3, λ_2_ = 2/3, ε = 0.1, the modified minimax design yields a design that requires modest increase of 1 to 3 additional subjects in 29% of cases, while the modified optimal design saves 1 to 13 subjects in 81% of cases for β = 0.2. Thus, the modified design approach provides investigators with an alternative when the sample sizes of Simon’s designs are severely unbalanced or the Type II error is unacceptably high after the first stage. The characteristics of the modified minimax and optimal designs for testing 20% and 15% improvement are presented in Supplementary Tables 1–6.

## SUPPLEMENTARY MATERIALS


