# Supplementary material for: Modified Simon’s minimax and optimal two-stage designs for single-arm phase II cancer clinical trials
Source: Oncotarget. 2019 Jul 2;10(42):4255–61. doi: 10.18632/oncotarget.26981 (PMC6611508; doi:10.18632/oncotarget.26981)
Supplement: Supplementary file 1 [file oncotarget-10-4255-s001.pdf]

Modified simon's minimax and optimal two-stage designs for single-arm phase II cancer clinical trials

SUPPLEMENTARY MATERIALS

(a) α=0.1 and β=0.1

P_0	P_1	sample size in total (*n*)												
		SM+0	+1	+2	+3	+4	+5	+6	+7	+8	+9	+10	+11	+12
0.05	0.25	M=SM	A	A		O=SO								
0.1	0.3	M=SM	A									O=SO		
0.15	0.35	M=SM	O=SO											
0.2	0.4	M=SM	O=SO											
0.25	0.45	M=SM	O=A				SO							
0.3	0.5	M≠SM			A				O=SO					
0.35	0.55	SM		M=A			O=SO							
0.4	0.6	M≠SM					O=SO							
0.45	0.65	M=SM				O=SO								
0.5	0.7	M=SM		A				O=SO						
0.55	0.75	SM	M=A		O=SO									
0.6	0.8	SM	M=A		SO			O						
0.65	0.85	M=SM			O=SO									
0.7	0.9	M=SM			O≠SO									
0.75	0.95	M=SM=SO=O												

(b) α=0.05 and β=0.2

P_0	P_1	sample size in total (*n*)												
		SM+0	+1	+2	+3	+4	+5	+6	+7	+8	+9	+10	+11	+12
0.05	0.25	SM	M=O=SO											
0.1	0.3	M=SM	O=A	A		SO(+3)								
0.15	0.35	M=O=SM					A	SO (+6)						
0.2	0.4	M=O=SM					A					SO (+10)		
0.25	0.45	M=SM	A		O		SO (+2)							
0.3	0.5	M=SM			O≠A				SO (+4)					
0.35	0.55	M=O=SM					SO (+5)							
0.4	0.6	SM		M=O=A					SO (+5)					
0.45	0.65	SM		M=O=A		SO (+2)								
0.5	0.7	M=SM		O=A				SO (+4)						
0.55	0.75	SM	M		O≠A				SO (+4)					
0.6	0.8	M=O=SM								SO (+8)				
0.65	0.85	SM	M=O=A		SO (+2)									
0.7	0.9	SM	SO	M=O										
0.75	0.95	SM		SO	M=O									

(c) α=0.05 and β=0.1

P_0	P_1	sample size in total (*n*)												
		SM+0	+1	+2	+3	+4	+5	+6	+7	+8	+9	+10	+11	+12
0.05	0.25	M=SM	A		O=A		SO							
0.1	0.3	M=SM	A	O=SO										
0.15	0.35	M=SM						O=SO						
0.2	0.4	M=SM				A					O=SO			
0.25	0.45	M=SM	A							O=SO				
0.3	0.5	M=SM			A							O=SO		
0.35	0.55	M≠SM			A			O=SO						
0.4	0.6	M=SM							A					O=SO
0.45	0.65	M=SM		A					O=SO					
0.5	0.7	M=SM				A				O=SO				
0.55	0.75	SM	M		A		O=SO							
0.6	0.8	M=SM		A						O=SO				
0.65	0.85	SM	M=A			O=SO								
0.7	0.9	M=SM				O=SO								
0.75	0.95	SM		M=O≠SO										

1. Abbreviation: M, the modified minimax design, O, the modified optimal design, SM, Simon's minimax design, SO, Simon's optimal design, A, the admissible design.
2. "=" indicates that they are identical but "≠" shows that they are not identical.
3. SO(+XX) shows that Simon's optimal design has "XX" additional samples, compared to the modified optimal design.

Supplementary Figure 1: Comparisons of the modified designs with $\gamma_1 = 1/3$, $\gamma_2 = 2/3$, and $\varepsilon = 0.1$ to Simon's and the admissible designs for $p_1 - p_0 = 0.2$.

(a) α=0.1 and β=0.1

P_0	P_1	sample size in total (n)																						
		SM+0	+1	+2	+3	+4	+5	+6	+7	+8	+9	+10	+11	+12	+13	+14	+15	+16	+17	+18	+19	+20	+21	+22
0.05	0.2	M=SM	A		A		SO	O																
0.1	0.25	M≠SM										O=SO												
0.15	0.3	M=SM	A	O=SO																				
0.2	0.35	M=SM					O=SO																	
0.25	0.4	M=SM				A				O=SO														
0.3	0.45	SM		M	A										O=SO									
0.35	0.5	M=SM			A			A			O=SO													
0.4	0.55	M=SM															O=SO							
0.45	0.6	SM	M=A	A		O=SO																		
0.5	0.65	M=SM		A				A						O=SO										
0.55	0.7	SM	M=A		A		O=SO																	
0.6	0.75	M≠SM		A					O=SO															
0.65	0.8	M=SM			O=SO																			
0.7	0.85	M=SM							O=SO															
0.75	0.9	M≠SM				A				O=SO														
0.8	0.95	SM=SO≠ M=O																						

(b) α=0.05 and β=0.2

P_0	P_1	sample size in total (n)																						
		SM+0	+1	+2	+3	+4	+5	+6	+7	+8	+9	+10	+11	+12	+13	+14	+15	+16	+17	+18	+19	+20	+21	+22
0.05	0.2	M=SM	O=A	SO (+1)																				
0.1	0.25	M=SM	O=A	A	SO (+2)																			
0.15	0.3	M=SM	O=A						SO (+6)															
0.2	0.35	M=SM					A			O	A										SO (+11)			
0.25	0.4	SM		M	A				O≠A				SO (+4)											
0.3	0.45	SM		M	O=A			A										SO (+13)						
0.35	0.5	SM		M			A			O≠A			SO (+3)											
0.4	0.55	SM	M	A							O					SO (+5)								
0.45	0.6	M=SM			A		A		SO			O												
0.5	0.65	SM	M=A		A		A		O		A						SO (+8)							
0.55	0.7	M=SM		A					O		SO (+2)													
0.6	0.75	M=SM					O≠SO																	
0.65	0.8	M=SM			A			O≠A						SO (+6)										
0.7	0.85	M=SM			O≠A							SO (+7)												
0.75	0.9	M=SM				A	O				SO (+4)													
0.8	0.95	SM=SO	M=O																					

(c) α=0.05 and β=0.1

P_0	P_1	sample size in total (n)																						
		SM+0	+1	+2	+3	+4	+5	+6	+7	+8	+9	+10	+11	+12	+13	+14	+15	+16	+17	+18	+19	+20	+21	+22
0.05	0.2	M≠SM	A	A	O=SO																			
0.1	0.25	M=SM	A	O=A									SO											
0.15	0.3	M=SM						A						A						O=SO				
0.2	0.35	M=SM	A					O=SO																
0.25	0.4	M≠SM	A			A				A								O=SO						
0.3	0.45	SM			M=A			A							A									O=SO
0.35	0.5	M=SM			A								O=SO											
0.4	0.55	M=SM					A					O=SO												
0.45	0.6	SM		A	M	A		A															O=SO	
0.5	0.65	M=SM		A		A								O=SO										
0.55	0.7	SM			M=A		A		A														O=SO	
0.6	0.75	SM	M=A		A								O=SO											
0.65	0.8	M≠SM			A			A						A						O=SO				
0.7	0.85	M=SM	A			A							SO			O								
0.75	0.9	M=SM					A				O=SO													
0.8	0.95	SM		M=O= SO																				

1. Abbreviation: M, the modified minimax design, O, the modified optimal design, SM, Simon's minimax design, SO, Simon's optimal design, A, the admissible design.
2. "=" indicates that they are identical but "≠" shows that they are not identical.
3. SO(+XX) shows that Simon's optimal design has "XX" additional samples, compared to the modified optimal design.

Supplementary Figure 2: Comparisons of the modified designs with γ_1 = 1/3, γ_2 = 2/3, and ε = 0.1 to Simon's and the admissible designs for $p_1 - p_0$ = 0.15.

Supplementary Table 1: The characteristics of the modified minimax and optimal designs for Δ = p_1 - p_0 = 0.2 and (α, β) = (0.1, 0.1)

p_0	p_1	α	β	Design method	r_1	n_1	r	n	EN_0	PET_0	PET_1	n_1/n (%)
0.05	0.25	0.1	0.1	Modified Minimax	0	13	2	20	16.4	0.513	0.024	65.0%
				Modified Optimal	0	9	2	24	14.5	0.630	0.075	37.5%
0.1	0.3	0.1	0.1	Modified Minimax	1	16	4	25	20.4	0.515	0.026	64.0%
				Modified Optimal	1	12	5	35	19.8	0.659	0.085	34.3%
0.15	0.35	0.1	0.1	Modified Minimax	2	17	7	32	24.2	0.520	0.033	53.1%
				Modified Optimal	3	19	7	33	23.4	0.684	0.059	57.6%
0.2	0.4	0.1	0.1	Modified Minimax	3	19	10	36	28.3	0.455	0.023	52.8%
				Modified Optimal	3	17	10	37	26.0	0.549	0.046	45.9%
0.25	0.45	0.1	0.1	Modified Minimax	5	23	13	39	31.5	0.468	0.019	59.0%
				Modified Optimal	3	15	13	40	28.5	0.461	0.042	37.5%
0.3	0.5	0.1	0.1	Modified Minimax	6	26	15	39	35.1	0.297	0.005	66.7%
				Modified Optimal	7	22	17	46	29.9	0.671	0.067	47.8%
0.35	0.55	0.1	0.1	Modified Minimax	7	21	19	44	31.7	0.536	0.038	47.7%
				Modified Optimal	7	20	20	47	30.8	0.601	0.058	42.6%
0.4	0.6	0.1	0.1	Modified Minimax	7	21	20	41	34.0	0.350	0.012	51.2%
				Modified Optimal	7	18	22	46	30.2	0.563	0.058	39.1%
0.45	0.65	0.1	0.1	Modified Minimax	9	21	22	41	30.8	0.512	0.031	51.2%
				Modified Optimal	9	20	24	45	30.2	0.591	0.053	44.4%
0.5	0.7	0.1	0.1	Modified Minimax	11	23	23	39	31.0	0.500	0.021	59.0%
				Modified Optimal	11	21	26	45	29.0	0.668	0.068	46.7%
0.55	0.75	0.1	0.1	Modified Minimax	13	23	25	39	28.8	0.636	0.041	59.0%
				Modified Optimal	10	18	26	41	27.0	0.609	0.057	43.9%
0.6	0.8	0.1	0.1	Modified Minimax	9	16	25	36	26.5	0.473	0.027	44.4%
				Modified Optimal	9	15	28	41	25.5	0.597	0.061	36.6%
0.65	0.85	0.1	0.1	Modified Minimax	8	13	23	31	22.0	0.499	0.034	41.9%
				Modified Optimal	10	15	25	34	21.7	0.648	0.062	44.1%
0.7	0.9	0.1	0.1	Modified Minimax	11	16	20	25	20.0	0.550	0.017	64.0%
				Modified Optimal	11	15	22	28	18.9	0.703	0.056	53.6%
0.75	0.95	0.1	0.1	Modified Minimax	6	8	16	19	12.0	0.633	0.057	42.1%
				Modified Optimal	6	8	16	19	12.0	0.633	0.057	42.1%

Supplementary Table 2: The characteristics of the modified minimax and optimal designs for Δ = p_1 - p_0 = 0.2 and (α, β) = (0.05, 0.2)

p_0	p_1	α	β	Design method	r_1	n_1	r	n	EN_0	PET_0	PET_1	n_1/n (%)
0.05	0.25	0.05	0.2	Modified Minimax	0	9	2	17	12.0	0.630	0.075	52.9%
				Modified Optimal	0	9	2	17	12.0	0.630	0.075	52.9%
0.1	0.3	0.05	0.2	Modified Minimax	1	15	5	25	19.5	0.549	0.035	60.0%
				Modified Optimal	1	12	5	26	16.8	0.659	0.085	46.2%
0.15	0.35	0.05	0.2	Modified Minimax	2	15	7	28	20.1	0.604	0.062	53.6%
				Modified Optimal	2	15	7	28	20.1	0.604	0.062	53.6%
0.2	0.4	0.05	0.2	Modified Minimax	4	18	10	33	22.3	0.716	0.094	54.5%
				Modified Optimal	4	18	10	33	22.3	0.716	0.094	54.5%
0.25	0.45	0.05	0.2	Modified Minimax	4	17	13	36	25.1	0.574	0.060	47.2%
				Modified Optimal	4	16	14	39	24.5	0.630	0.085	41.0%
0.3	0.5	0.05	0.2	Modified Minimax	6	19	16	39	25.7	0.666	0.084	48.7%
				Modified Optimal	4	14	17	42	25.6	0.584	0.090	33.3%
0.35	0.55	0.05	0.2	Modified Minimax	8	21	18	39	26.3	0.706	0.091	53.8%
				Modified Optimal	8	21	18	39	26.3	0.706	0.091	53.8%
0.4	0.6	0.05	0.2	Modified Minimax	7	17	21	41	25.6	0.641	0.092	41.5%
				Modified Optimal	7	17	21	41	25.6	0.641	0.092	41.5%
0.45	0.65	0.05	0.2	Modified Minimax	8	17	23	41	25.1	0.663	0.099	41.5%
				Modified Optimal	8	17	23	41	25.1	0.663	0.099	41.5%
0.5	0.7	0.05	0.2	Modified Minimax	12	23	23	37	27.7	0.661	0.055	62.2%
				Modified Optimal	8	16	24	39	25.2	0.598	0.074	41.0%
0.55	0.75	0.05	0.2	Modified Minimax	11	20	25	37	27.0	0.586	0.041	54.1%
				Modified Optimal	7	13	26	39	24.1	0.573	0.080	33.3%
0.6	0.8	0.05	0.2	Modified Minimax	8	13	25	35	20.8	0.647	0.099	37.1%
				Modified Optimal	8	13	25	35	20.8	0.647	0.099	37.1%
0.65	0.85	0.05	0.2	Modified Minimax	7	11	24	31	19.5	0.574	0.069	35.5%
				Modified Optimal	7	11	24	31	19.5	0.574	0.069	35.5%
0.7	0.9	0.05	0.2	Modified Minimax	8	11	23	28	16.3	0.687	0.090	39.3%
				Modified Optimal	8	11	23	28	16.3	0.687	0.090	39.3%
0.75	0.95	0.05	0.2	Modified Minimax	8	10	20	23	13.2	0.756	0.086	43.5%
				Modified Optimal	8	10	20	23	13.2	0.756	0.086	43.5%

Supplementary Table 3: The characteristics of the modified minimax and optimal designs for Δ = p_1 - p_0 = 0.2 and (α, β) = (0.05, 0.1)

p_0	p_1	α	β	Design method	r_1	n_1	r	n	EN_0	PET_0	PET_1	n_1/n (%)
0.05	0.25	0.05	0.1	Modified Minimax	0	15	3	25	20.4	0.463	0.013	60.0%
				Modified Optimal	0	10	3	28	17.2	0.599	0.056	35.7%
0.1	0.3	0.05	0.1	Modified Minimax	2	22	6	33	26.2	0.620	0.021	66.7%
				Modified Optimal	2	18	6	35	22.5	0.734	0.060	51.4%
0.15	0.35	0.05	0.1	Modified Minimax	3	23	9	38	29.9	0.540	0.018	60.5%
				Modified Optimal	3	19	10	44	26.9	0.684	0.059	43.2%
0.2	0.4	0.05	0.1	Modified Minimax	5	24	13	45	31.2	0.656	0.040	53.3%
				Modified Optimal	4	19	15	54	30.4	0.673	0.070	35.2%
0.25	0.45	0.05	0.1	Modified Minimax	6	26	17	49	37.1	0.515	0.018	53.1%
				Modified Optimal	6	22	19	57	32.5	0.699	0.071	38.6%
0.3	0.5	0.05	0.1	Modified Minimax	7	24	21	53	36.6	0.565	0.032	45.3%
				Modified Optimal	8	24	24	63	34.7	0.725	0.076	38.1%
0.35	0.55	0.05	0.1	Modified Minimax	10	34	24	53	47.1	0.313	0.002	64.2%
				Modified Optimal	7	20	26	59	35.6	0.601	0.058	33.9%
0.4	0.6	0.05	0.1	Modified Minimax	12	29	27	54	38.1	0.637	0.033	53.7%
				Modified Optimal	11	25	32	66	36.0	0.732	0.078	37.9%
0.45	0.65	0.05	0.1	Modified Minimax	14	31	30	54	40.6	0.581	0.019	57.4%
				Modified Optimal	11	23	33	61	34.9	0.687	0.068	37.7%
0.5	0.7	0.05	0.1	Modified Minimax	14	27	32	53	36.1	0.649	0.036	50.9%
				Modified Optimal	13	24	36	61	34.0	0.729	0.074	39.3%
0.55	0.75	0.05	0.1	Modified Minimax	15	28	33	50	38.7	0.513	0.011	56.0%
				Modified Optimal	10	18	35	54	32.1	0.609	0.057	33.3%
0.6	0.8	0.05	0.1	Modified Minimax	15	26	32	45	35.9	0.479	0.008	57.8%
				Modified Optimal	12	19	37	53	29.5	0.692	0.068	35.8%
0.65	0.85	0.05	0.1	Modified Minimax	12	18	31	41	26.2	0.645	0.042	43.9%
				Modified Optimal	10	15	33	44	25.2	0.648	0.062	34.1%
0.7	0.9	0.05	0.1	Modified Minimax	13	18	26	32	22.7	0.667	0.028	56.3%
				Modified Optimal	11	15	29	36	21.2	0.703	0.056	41.7%
0.75	0.95	0.05	0.1	Modified Minimax	12	15	24	28	18.1	0.764	0.036	53.6%
				Modified Optimal	12	15	24	28	18.1	0.764	0.036	53.6%

Supplementary Table 4: The characteristics of the modified minimax and optimal designs for Δ = p_1 - p_0 = 0.15 and (α, β) = (0.1, 0.1)

p_0	p_1	α	β	Design method	r_1	n_1	r	n	EN_0	PET_0	PET_1	n_1/n (%)
0.05	0.2	0.1	0.1	Modified Minimax	0	18	3	32	26.4	0.397	0.018	56.3%
				Modified Optimal	1	19	3	38	23.7	0.755	0.083	50.0%
0.1	0.25	0.1	0.1	Modified Minimax	1	23	6	40	34.6	0.315	0.012	57.5%
				Modified Optimal	2	21	7	50	31.2	0.648	0.075	42.0%
0.15	0.3	0.1	0.1	Modified Minimax	5	34	11	53	41.7	0.597	0.033	64.2%
				Modified Optimal	3	23	11	55	37.7	0.54	0.054	41.8%
0.2	0.35	0.1	0.1	Modified Minimax	6	33	15	58	45.5	0.5	0.028	56.9%
				Modified Optimal	5	27	16	63	43.6	0.539	0.051	42.9%
0.25	0.4	0.1	0.1	Modified Minimax	9	39	20	64	52.1	0.476	0.02	60.9%
				Modified Optimal	7	29	22	72	48.1	0.557	0.057	40.3%
0.3	0.45	0.1	0.1	Modified Minimax	13	45	26	71	57.8	0.509	0.02	63.4%
				Modified Optimal	9	30	29	82	51.4	0.589	0.069	36.6%
0.35	0.5	0.1	0.1	Modified Minimax	14	43	30	72	59.3	0.437	0.016	59.7%
				Modified Optimal	12	34	33	81	53.2	0.592	0.061	42.0%
0.4	0.55	0.1	0.1	Modified Minimax	18	45	34	73	57.2	0.564	0.031	61.6%
				Modified Optimal	16	38	40	88	54.5	0.67	0.076	43.2%
0.45	0.6	0.1	0.1	Modified Minimax	22	50	39	75	62.5	0.502	0.016	66.7%
				Modified Optimal	14	32	40	78	54.2	0.517	0.046	41.0%
0.5	0.65	0.1	0.1	Modified Minimax	19	40	41	72	58.0	0.437	0.017	55.6%
				Modified Optimal	18	35	47	84	53.0	0.632	0.068	41.7%
0.55	0.7	0.1	0.1	Modified Minimax	16	31	44	71	54.2	0.419	0.024	43.7%
				Modified Optimal	19	34	46	75	50.1	0.606	0.057	45.3%
0.6	0.75	0.1	0.1	Modified Minimax	22	39	43	64	54.5	0.381	0.009	60.9%
				Modified Optimal	21	34	47	71	47.1	0.646	0.061	47.9%
0.65	0.8	0.1	0.1	Modified Minimax	22	33	43	60	42.6	0.643	0.051	55.0%
				Modified Optimal	20	30	45	63	41.8	0.642	0.061	47.6%
0.7	0.85	0.1	0.1	Modified Minimax	15	22	40	52	36.8	0.506	0.037	42.3%
				Modified Optimal	14	20	45	59	36.2	0.584	0.067	33.9%
0.75	0.9	0.1	0.1	Modified Minimax	19	26	33	40	33.2	0.485	0.012	65.0%
				Modified Optimal	12	16	39	48	29.0	0.595	0.068	33.3%
0.8	0.95	0.1	0.1	Modified Minimax	13	16	27	31	21.3	0.648	0.043	51.6%
				Modified Optimal	13	16	27	31	21.3	0.648	0.043	51.6%

Supplementary Table 5: The characteristics of the modified minimax and optimal designs for Δ = p_1 - p_0 = 0.15 and (α, β) = (0.05, 0.2)

p_0	p_1	α	β	Design method	r_1	n_1	r	n	EN_0	PET_0	PET_1	n_1/n (%)
0.05	0.2	0.05	0.2	Modified Minimax	0	13	3	27	19.8	0.513	0.055	48.1%
				Modified Optimal	0	11	3	28	18.3	0.569	0.086	39.3%
0.1	0.25	0.05	0.2	Modified Minimax	2	22	7	40	28.8	0.62	0.061	55.0%
				Modified Optimal	1	15	7	41	26.7	0.549	0.08	36.6%
0.15	0.3	0.05	0.2	Modified Minimax	3	23	11	48	34.5	0.54	0.054	47.9%
				Modified Optimal	3	21	11	49	31.9	0.611	0.086	42.9%
0.2	0.35	0.05	0.2	Modified Minimax	6	31	15	53	40.4	0.571	0.046	58.5%
				Modified Optimal	4	21	17	61	37.6	0.586	0.092	34.4%
0.25	0.4	0.05	0.2	Modified Minimax	10	40	21	62	49.2	0.584	0.035	64.5%
				Modified Optimal	6	24	22	67	40.9	0.607	0.096	35.8%
0.3	0.45	0.05	0.2	Modified Minimax	9	31	26	67	47.5	0.542	0.052	46.3%
				Modified Optimal	7	24	26	68	43.2	0.565	0.086	35.3%
0.35	0.5	0.05	0.2	Modified Minimax	14	42	30	68	55.5	0.481	0.022	61.8%
				Modified Optimal	10	28	32	74	45.7	0.616	0.092	37.8%
0.4	0.55	0.05	0.2	Modified Minimax	15	40	35	71	57.4	0.44	0.02	56.3%
				Modified Optimal	12	29	38	79	47.1	0.637	0.099	36.7%
0.45	0.6	0.05	0.2	Modified Minimax	19	42	38	70	53.9	0.576	0.038	60.0%
				Modified Optimal	14	30	43	80	47.8	0.645	0.097	37.5%
0.5	0.65	0.05	0.2	Modified Minimax	20	41	41	69	55.0	0.5	0.024	59.4%
				Modified Optimal	15	29	44	75	45.4	0.644	0.098	38.7%
0.55	0.7	0.05	0.2	Modified Minimax	20	35	43	67	45.8	0.662	0.073	52.2%
				Modified Optimal	14	25	47	74	43.8	0.616	0.098	33.8%
0.6	0.75	0.05	0.2	Modified Minimax	18	30	43	62	43.8	0.569	0.051	48.4%
				Modified Optimal	14	23	46	67	40.1	0.612	0.096	34.3%
0.65	0.8	0.05	0.2	Modified Minimax	20	31	41	55	41.9	0.545	0.033	56.4%
				Modified Optimal	16	24	45	61	37.2	0.642	0.089	39.3%
0.7	0.85	0.05	0.2	Modified Minimax	16	23	39	49	34.4	0.56	0.046	46.9%
				Modified Optimal	16	22	41	52	31.4	0.687	0.1	42.3%
0.75	0.9	0.05	0.2	Modified Minimax	17	22	33	39	27.5	0.677	0.062	56.4%
				Modified Optimal	14	18	37	44	25.9	0.694	0.098	40.9%
0.8	0.95	0.05	0.2	Modified Minimax	13	16	27	30	20.9	0.648	0.043	53.3%
				Modified Optimal	13	16	27	30	20.9	0.648	0.043	53.3%

Supplementary Table 6: The characteristics of the modified minimax and optimal designs for Δ = p_1 - p_0 = 0.15 and (α, β) = (0.05, 0.1)

p_0	p_1	α	β	Design method	r_1	n_1	r	n	EN_0	PET_0	PET_1	n_1/n (%)
0.05	0.2	0.05	0.1	Modified Minimax	0	23	4	38	33.4	0.307	0.006	60.5%
				Modified Optimal	1	21	4	41	26.7	0.717	0.058	51.2%
0.1	0.25	0.05	0.1	Modified Minimax	3	31	9	55	40.0	0.624	0.031	56.4%
				Modified Optimal	3	28	9	57	36.9	0.695	0.055	49.1%
0.15	0.3	0.05	0.1	Modified Minimax	6	42	14	64	51.8	0.555	0.015	65.6%
				Modified Optimal	5	30	17	82	45.1	0.711	0.077	36.6%
0.2	0.35	0.05	0.1	Modified Minimax	8	42	21	77	58.4	0.531	0.019	54.5%
				Modified Optimal	8	37	22	83	51.4	0.686	0.059	44.6%
0.25	0.4	0.05	0.1	Modified Minimax	11	52	27	83	73.0	0.323	0.003	62.7%
				Modified Optimal	10	37	31	99	56.2	0.691	0.072	37.4%
0.3	0.45	0.05	0.1	Modified Minimax	14	46	34	91	64.1	0.597	0.032	50.5%
				Modified Optimal	13	40	40	110	60.8	0.703	0.075	36.4%
0.35	0.5	0.05	0.1	Modified Minimax	16	46	40	94	67.4	0.555	0.027	48.9%
				Modified Optimal	16	43	44	105	62.7	0.683	0.063	41.0%
0.4	0.55	0.05	0.1	Modified Minimax	24	62	45	94	78.9	0.472	0.007	66.0%
				Modified Optimal	19	45	49	104	64.0	0.679	0.058	43.3%
0.45	0.6	0.05	0.1	Modified Minimax	26	58	52	98	76.3	0.544	0.014	59.2%
				Modified Optimal	19	40	60	116	64.0	0.684	0.074	34.5%
0.5	0.65	0.05	0.1	Modified Minimax	28	57	54	93	75.0	0.5	0.01	61.3%
				Modified Optimal	22	42	60	105	62.3	0.678	0.062	40.0%
0.55	0.7	0.05	0.1	Modified Minimax	26	47	58	92	66.2	0.574	0.023	51.1%
				Modified Optimal	22	38	68	110	59.8	0.697	0.076	34.5%
0.6	0.75	0.05	0.1	Modified Minimax	29	48	58	85	63.6	0.578	0.018	56.5%
				Modified Optimal	21	34	64	95	55.6	0.646	0.061	35.8%
0.65	0.8	0.05	0.1	Modified Minimax	29	46	55	75	62.1	0.445	0.006	61.3%
				Modified Optimal	21	31	67	93	50.3	0.689	0.075	33.3%
0.7	0.85	0.05	0.1	Modified Minimax	33	44	53	68	48.5	0.811	0.057	64.7%
				Modified Optimal	23	31	63	82	43.5	0.755	0.082	37.8%
0.75	0.9	0.05	0.1	Modified Minimax	19	25	45	54	36.0	0.622	0.033	46.3%
				Modified Optimal	18	23	52	63	34.3	0.717	0.073	36.5%
0.8	0.95	0.05	0.1	Modified Minimax	16	19	37	42	24.4	0.763	0.067	45.2%
				Modified Optimal	16	19	37	42	24.4	0.763	0.067	45.2%
